# Microvascular brain damage in middle-aged women with a history of migraine with aura and/or ischemic stroke

**DOI:** 10.1177/17474930251389728

**Published:** 2025-10-15

**Authors:** Nelleke van der Weerd, Annelise E Wilms, Hendrikus JA van Os, Ghislaine Holswilder, Katie M Linstra, Erik W van Zwet, Arn MJM van den Maagdenberg, Antoinette MaassenvandenBrink, Mark C Kruit, Gisela M Terwindt, Marieke JH Wermer

**Affiliations:** 1 Department of Neurology, Leiden University Medical Centre, Leiden, The Netherlands; 2 Department of Human Genetics, Leiden University Medical Centre, Leiden, The Netherlands; 3 Department of Public Health and Primary Care, Leiden University Medical Centre, Leiden, The Netherlands; 4 Department of Medical Statistics, Leiden University Medical Centre, Leiden, The Netherlands; 5 Department of Internal Medicine, Erasmus University Medical Centre, Rotterdam, The Netherlands; 6 Department of Radiology, Leiden University Medical Centre, Leiden, The Netherlands; 7 Department of Neurology, Groningen University Medical Centre, Groningen, The Netherlands

**Keywords:** Ischemic stroke, migraine with aura, microvascular damage, white matter hyperintensities, enlarged perivascular spaces

## Abstract

**Background:**

Both patients with migraine with aura (MA) and patients with ischemic stroke have an increased risk of white matter hyperintensities (WMH) indicating structural microvascular brain damage. It is unclear whether other signs of microvascular damage are also more abundant in these patients, and whether patients with both conditions are more severely affected.

**Methods:**

We included middle-aged women with a history of MA, ischemic stroke, or both, as well as age-matched female control participants without any neurological disease, from two cross-sectional MRI studies (CREW and WHISPER). We assessed WMH, enlarged perivascular spaces, cerebral microbleeds, lacunes, cortical superficial siderosis, parenchymal volume, and cortical atrophy, according to STRIVE criteria. A total small vessel disease (SVD) burden score was determined. We performed regression analyses to assess the association between a history of MA, stroke, or both and the different MRI markers, adjusted for vascular risk factors.

**Results:**

We included 207 women (mean age: 51 years): 39 with MA, 67 with stroke, 62 with both MA and stroke, and 39 controls. MA was not associated with increased microvascular damage compared with controls. Stroke patients had more cerebellar WMH (OR = 7.9, 95% CI = 0.9–73.6), more cortical atrophy (β = 0.2, 95% CI = 0.0–0.4), and a lower parenchymal volume (β = −16.1, 95% CI = −30.7 to −1.4) than controls. There was no difference in the frequency of any of the SVD markers on 3 Tesla (3T)-MRI in patients with stroke with or without migraine.

**Conclusion:**

In our study, markers of microvascular cerebral damage were infrequent in middle-aged women with MA and healthy controls, while stroke was associated with more cerebellar WMH, decreased parenchymal volume, and cortical atrophy. We found no (supra-)additive effect of a history of migraine on the extent of microvascular brain damage in women with stroke.

## Graphical abstract

**Graphical abstract:**
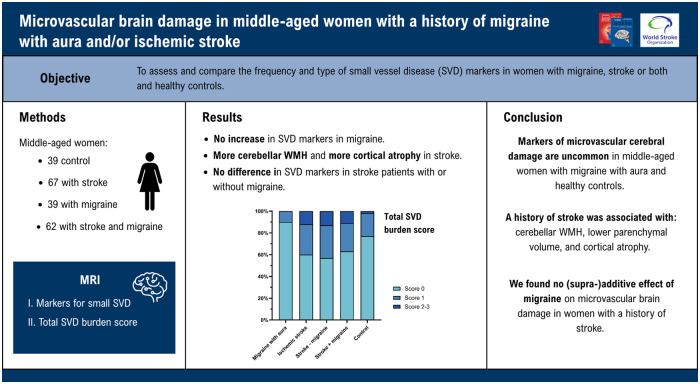

## Introduction

White matter hyperintensities (WMH) are seen at a relatively young age in both patients with migraine with aura (MA) and patients with ischemic stroke, even in the absence of vascular risk factors.^1,2^ Moreover, MA increases risk of ischemic stroke, especially in women.^2^ The risk of WMH in patients with MA is sex-dependent and higher for women than for men.^3^ The presence of WMH suggests more widespread damage of the cerebral microvasculature. Microvascular damage could lead to more cognitive decline or dementia later in life, showing the importance of understanding WMH in these patients.^4^ With current 3 Tesla (3T) MRI techniques, other markers for microvascular brain damage such as cerebral microbleeds, micro-infarcts, cortical superficial siderosis, enlarged perivascular spaces (EPVS), lacunes, and cortical atrophy, can be detected in great detail.^5,6^

Although some research has been done on individual MRI markers of microvascular brain damage in small cohorts of patients with migraine or ischemic stroke, it remains unexplored whether the pathophysiology behind these lesions is similar for both diseases.^1,2,7–14^ Also, no studies have investigated microvascular damage in patients with both migraine and ischemic stroke, compared with patients with only one of the two diseases. Thus, it is unclear whether the frequency and type of microvascular damage is similar or distinct in migraine and ischemic stroke, and whether there is an additional, or even supra-additive (indicating a greater risk of microvascular damage than expected), impact of migraine on microvascular damage in women with ischemic stroke. We, therefore, performed an exploratory study investigating multiple markers of microvascular damage on 3T MRI in middle-aged women with MA, women with a history of ischemic stroke, as well as women with both diseases.

## Methods

### Study population

We recruited participants from two cross-sectional studies with identical protocols: (1) The “White Matter Lesions in Young to Middle Aged Women with Stroke, Preeclampsia and Migraine: A 3 and 7 Tesla Study” (WHISPER) and (2) the Cardiovascular Risk Profile in Women—Microvascular Status (CREW-MIST). Both studies were performed at the Leiden University Medical Center (LUMC) between 2016 and 2022. The CREW-MIST study investigated women with a history of ischemic stroke with or without (a history of) migraine; the WHISPER study included women with MA without ischemic stroke and a control group.

Four groups of women between 40 and 60 years of age with (1) ischemic stroke, (2) MA, (3) both ischemic stroke and migraine (with and without aura) were included, and compared with (4) controls without neurological diseases. Women with ischemic stroke were recruited through the (outpatient) clinic of the Neurology Department of the LUMC and by recruitment via advertisement from the Netherlands Heart Foundation. Ischemic stroke was defined as acute neurological deficits lasting >24 h with corresponding lesions seen on CT and/or MRI. Stroke was divided into five subtypes following the TOAST classification: (1) large artery atherosclerosis (LAA), (2) cardio-embolism (CE), (3) small vessel occlusion (SVO), (4) undetermined, and (5) other cause.^15^ For classification, we used clinical findings, neuroimaging reports (CT/MRI), and additional diagnostic tests. Details on the location of ischemic stroke were extracted from medical records. Women with MA were recruited from the Leiden University Medical Center Migraine Neuroanalysis program (LUMINA) cohort.^16^ Migraine was classified according to the International Classification of Headache Disorder-3 criteria.^17^ Migraine patients included in the WHISPER study were required to have an active form of migraine with an attack frequency of one or more attacks per month, with at least one attack with aura in the previous 12 months. Female control participants without stroke or MA were recruited via an advertisement of the Netherlands Heart Foundation. For all groups, women were excluded when they had a retinal, spinal, or venous infarction, any other neurological disorder, any serious illness compromising study participation, or any contraindication for MRI.

Participants filled in a questionnaire on general demographics, health history, obstetric history, vascular risk factors, and medication use. On the research day, a 3T MRI was performed. The study protocols of WHISPER (P18.130) and CREW-MIST (P15.384) were approved by the Medical Ethics Committee Leiden—Den Haag—Delft (METC-LDD), and all participants gave written informed consent before study participation.

### MRI markers of microvascular brain damage

All MRI markers were assessed by two trained researchers (NW and AW) independent of each other, following the STandards for Reporting Vascular changes on nEuroimaging (STRIVE) criteria.^18^ A consensus meeting was held for divergent scores. When no consensus could be reached, an experienced neuroradiologist (MK) was consulted to obtain a final decision. Researchers were blinded to migraine status and clinical data. In ischemic stroke patients, WMH, cerebral microbleeds, and cerebral volume were only scored/measured in the unaffected hemisphere. Further details on the MRI protocol and scoring are provided in the Supplemental Methods.

WMH were considered to be present if hyperintense on FLAIR and divided into periventricular (PV-WMH), deep (D-WMH), and cerebellar. The Fazekas score was graded based on size and confluence.^19^ Cerebellar WMH were only scored as present or absent.

EPVS were defined as fluid-filled spaces following vessels through gray or white matter. They were distinguished as being round or ovoid with a diameter <3 mm when imaged perpendicular to the vessel. EPVS were counted in the basal ganglia, centrum semiovale, and midbrain on T2-weighted scans following a qualitative rating scale.^20^

Cerebral microbleeds were counted on susceptibility weighted imaging (SWI) per region (infratentorial, deep, white matter, cortical). Microbleeds were defined as round or ovoid areas of signal void with associated blooming, generally 2 to 5 mm but up to 10 mm.^5^

Lacunes were counted on FLAIR images in the basal ganglia, thalamus, capsula interna, and white matter. Lacunes were defined as round or ovoid, fluid-filled hypointense cavities with a hyperintense rim, between 3 and 15 mm in diameter.^5^

Cortical superficial siderosis (cSS) was scored on SWI images following two scoring systems: focal or disseminated. cSS was rated as focal when it was restricted to three or fewer sulci and disseminated when it was affecting four or more sulci.^21^

Cerebral volume and cortical atrophy were determined using a fully automated tool cNeuro (version 1.11.0; Combinostics Ltd, Tampere, Finland). Cortical atrophy was assessed according to the Pasquier visual rating scale (range: 0–3)^22^ and was computationally estimated based on the concentration of gray matter. Cerebral volume was multiplied by two for ischemic stroke to estimate total cerebral volume.

Total small vessel disease (SVD) burden score (range: 0–4), a previously validated score for microvascular damage, was calculated by assigning one point for each of the following aspects: (1) perivascular WMH Fazekas score of 3 and/or deep WMH Fazekas score of 2–3, (2) presence of lacunes, (3) presence of microbleeds, and (4) presence of EPVS in basal ganglia with a score ⩾2.^23^

### Statistical analysis

Frequencies of the markers are shown as proportions. We performed logistic regression analysis for markers with a binary outcome and ordinal logistic regression analysis for those with an ordinal outcome. Multiple linear regression was used for brain and cortical volumes, and computed global atrophy. MA and ischemic stroke were compared with controls, followed by subgroup analyses comparing stroke patients with and without migraine. We adjusted for age, smoking (ever), hypertension, diabetes, and hypercholesterolemia. Due to the exploratory nature of this study, we did not adjust for multiple testing.^24^ All statistical analyses were performed in R 4.2.1 using the *stats* package (4.2.1) for linear and logistic regression, and the *MASS* package (7.3–58) for ordinal logistic regression.

## Results

In total, 207 women were included in this study: 39 with MA, 129 with a history of ischemic stroke (67 without migraine and 62 with migraine (30 with MA and 32 with migraine without aura (MO))), and 39 controls. From the 129 women with ischemic stroke, 30 (23%) had LAA, 10 (8%) had CE, 22 (17%) had SVO, 50 (39%) had a stroke of undetermined origin, and the remaining 14 (11%) had another etiology (Table 1). The mean age was 51 ± 5 years across all groups. On average, women with ischemic stroke participated 4.9 ± 4.5 years after their stroke in this study. Women with ischemic stroke more often had hypertension and hypercholesterolemia compared with women with MA or controls.

**Table 1.** Characteristics of the participants.

	**Migraine with aura (*n*** **=** **39)**	**Ischemic stroke (*n*** **=** **129)**	*Stroke—migraine (n* *=* *67)*	*Stroke* *+* *migraine (n* *=* *62)*	**Controls (*n*** **=** **39)**
Age at visit, years **±** SD	51 ± 5	51 ± 5	*51* *±* *6*	*51* *±* *5*	52 ± 5
Age at stroke, years ± SD	N/A	46 ± 6	*47* *±* *6*	*45* *±* *6*	N/A
**Migraine**, ***n*** **(%)**					
MA	39 (100)	N/A	*N/A*	*30 (48)*	N/A
MO	0 (0)			*32 (52)*	
*****Attack frequency (per year)*****					
1–6	0 (0)			*28 (45)*	
7–12	9 (23)			*10 (16)*	
13–54	30 (77)			*18 (29)*	
>54	0 (0)			*6 (10)*	
**Vascular risk factors, ***n*** (%)**					
Hypertension	12 (31)	81 (63)	*40 (60)*	*41 (66)*	9 (23)
Hypercholesterolemia	7 (18)	102 (79)	*53 (79)*	*49 (79)*	2 (5)
TIA	0 (0)	10 (8)	*5 (8)*	*5 (8)*	0 (0)
Current smoking	4 (10)	13 (10)	*8 (12)*	*5 (8)*	2 (5)
Ever smoking	9 (23)	58 (45)	*37 (55)*	*21 (34)*	13 (33)
Pack years **±** SD	10.0 ± 18.6	11.4 ± 13.3	*15.1* *±* *14.1*	*7.2* *±* *11.0*	4.0 ± 12.5
Alcohol > 2 units per day	0 (0)	2 (2)	*2 (3)*	*0 (0)*	0 (0)
Diabetes mellitus	1 (3)	7 (5)	*3 (4)*	*4 (6)*	0 (0)
BMI **±** SD	25.4 ± 4.1	27.2 ± 4.8	*27.6* *±* *5.1*	*26.7* *±* *4.5*	25.2 ± 4.0
**TOAST**, ***n*** (%)*					
LAA	N/A	30 (23)	*19 (28)*	*11 (18)*	N/A
CE		10 (8)	*6 (9)*	*4 (6)*	
SVO		22 (17)	*11 (16)*	*11 (18)*	
Other cause		14 (11)	*11 (16)*	*3 (5)*	
Undetermined		50 (39)	*19 (28)*	*31 (50)*	
**Other**					
PCOS	3 (8)	1 (1)	*0 (0)*	*1 (2)*	1 (3)
PE	0 (0)	5 (4)	*2 (3)*	*3 (5)*	0 (0)
Postmenopausal	19 (49)	56 (43)	*29 (48)*	*27 (44)*	18 (46)
Postmenopausal age ± SD	46 ± 7	48 ± 6	*47* *±* *6*	*48* *±* *6*	50 ± 4
Oral contraceptives use	5 (13)	3 (2)	*2 (3)*	*1 (2)*	2 (5)
**Anticoagulant use**					
Antiplatelets	3 (8)	115 (89)	*55 (82)*	*60 (97)*	0 (0)
Vitamin-K antagonist	0 (0)	10 (8)	*7 (10)*	*3 (5)*	0 (0)
Heparin	1 (3)	15 (12)	*10 (15)*	*5 (8)*	0 (0)
**Migraine medication use**					
NSAIDS	24 (62)	N/A		*40 (65)*	
Ergotamine use	1 (3)		*N/A*	*2 (3)*	N/A
Triptan use	27 (69)			*11 (18)*	

MA: Migraine with aura; MO: migraine without aura; TIA: transient ischemic attack; LAA: large artery atherosclerosis; CE, cardio-embolism; SVO: small vessel occlusion; PCOS: polycystic ovary syndrome; PE: pre-eclampsia; NSAIDS: non-steroidal anti-inflammatory drug.

*Italic:* the subgroups of women with stroke without migraine and women with stroke and migraine.

* Three missings.

### Microvascular brain damage MRI markers

In all groups, most participants had a WMH Fazekas score (periventricular and deep) of 1 (Table 2). Cerebellar WMH were present in 4 (10%) participants with MA, 23 (18%) participants with ischemic stroke, and 1 (3%) control (Table 2). We found no difference in WMH Fazekas score (periventricular and deep) in MA compared with controls (Supplemental Table 1). Women with stroke had no increase in periventricular or deep WMH compared with controls, but had a higher risk of cerebellar WMH, although this increased risk was only statistically significant for women with stroke without migraine (overall: OR = 8.3, 95% CI = 0.9–78.2; stroke without migraine: OR = 11.7, 95% CI = 1.2–111.9; stroke with migraine: OR = 8.8, 95% CI = 0.9–85.9, Supplemental Table 1). There were no differences between stroke subgroups (*p* = 0.600, Supplemental Table 2).

**Table 2.** Microvascular brain damage.

	**Migraine with aura (*n*** **=** **39)**	**Ischemic stroke (*n*** **=** **129)**	*Stroke—migraine (n* *=* *67)*	*Stroke* *+* *migraine (n* *=* *62)*	**Control (*n*** **=** **39)**
**Fazekas score**					
WMH-PV*, ***n*** (%)					
0	4 (10)	13 (10)	*7 (10)*	*6 (10)*	3 (8)
1	31 (80)	94 (73)	*46 (69)*	*48 (77)*	35 (90)
2–3	4 (10)	21 (16)	*13 (19)*	*8 (13)*	1 (2)
WMH-D*, ***n*** (%)					
0	8 (21)	29 (22)	*17 (25)*	*12 (19)*	11 (28)
1	29 (74)	87 (67)	*40 (60)*	*47 (76)*	24 (62)
2–3	2 (5)	12 (9)	*9 (13)*	*3 (5)*	4 (10)
WMH cerebellar*, ***n*** (%)	4 (10)	23 (18)	*12 (18)*	*11 (18)*	1 (3)
**EPVS** ^†^					
**Basal ganglia, ***n*** (%)**					
0	0 (0)	2 (2)	*1 (2)*	*1 (2)*	0 (0)
1	37 (95)	98 (76)	*54 (81)*	*44 (71)*	39 (100)
2–4	1 (3)	26 (20)	*11 (16)*	*15 (24)*	0 (0)
**Centrum semiovale, ***n*** (%)**					
0	0 (0)	2 (2)	*1 (2)*	*1 (2)*	0 (0)
1	12 (31)	27 (21)	*15 (22)*	*12 (19)*	11 (28)
2–4	23 (59)	94 (73)	*48 (72)*	*46 (74)*	28 (72)
**Midbrain, ***n*** (%)**	26 (67)	89 (69)	*48 (72)*	*41 (66)*	25 (64)
Microbleeds**, ***n*** (%)	1 (3)	20 (16)	*12 (18)*	*8 (13)*	3 (8)
Lacunes*, ***n*** (%)	0 (0)	19 (15)	*11 (16)*	*8 (13)*	3 (8)
cSS**, ***n*** (%)					
Focal	0 (0)	3 (2)	*1 (1)*	*2 (3)*	0 (0)
Disseminated	0 (0)	0 (0)	*0 (0)*	*0 (0)*	0 (0)
**Brain volume and atrophy** ^††^					
Parenchymal volume, mL ± SD	1170.7 ± 25.8	1156.8 ± 32.4	*1152.2* *±* *29.6*	*1161.3* *±* *34.7*	1172.2 ± 18.0
Cortical volume, mL ± SD	509.9 ± 46.2	527.7 ± 34.4	*531.6* *±* *41.2*	*523.3* *±* *24.6*	552.2 ± 14.9
Cortical atrophy	0.0 ± 0.0	0.2 ± 0.5	*0.2* *±* *0.5*	*0.2* *±* *0.5*	0.0 ± 0.0
**Total SVD burden, ***n*** (%)**					
0	35 (90)	77 (60)	*38 (57)*	*39 (63)*	30 (77)
1	4 (10)	36 (28)	*20 (30)*	*16 (26)*	8 (21)
2–4	0 (0)	16 (12)	*9 (13)*	*7 (11)*	1 (2)

* Missing due to missing FLAIR MRI scan: WMH-PV (1), WMH-D (1), lacunes (1), and 15 missing WMH cerebellar due to 14 cerebellar infarctions.

** Missing due to missing SWI MRI scan or moving artifacts: microbleeds (6).

† Missing due to missing T2 MRI scan or moving artifacts: BG (4), CS (10), and midbrain (2).

†† Missing due to missing T1 MRI scan (2), atrophy data missing due to infarctions in both hemispheres (6). *Italic:* the subgroups of women with stroke without migraine and women with stroke and migraine.

The majority of the participants had an EPVS score of 1 in basal ganglia, an EPVS score of 2 to 4 in centrum semiovale, and had more than 1 EPVS in midbrain (Table 2). We found no difference in EPVS in basal ganglia, centrum semiovale, and midbrain in stroke or MA compared with controls and in women with stroke with and without migraine (Supplemental Tables 1 and 2).

Twenty-four (12%) of the participants had cerebral microbleeds; 1 (3%) MA, 20 (16%) ischemic stroke (without migraine, *n* = 12; with migraine, *n* = 8), and 3 (8%) controls. Most (58%) of participants with cerebral microbleeds had a single cerebral microbleed. Average number of microbleeds is shown in Supplemental Table 3. Women with stroke had no more cerebral microbleeds compared with controls, and there was no difference in cerebral microbleed presence for women with stroke with and without migraine.

Twenty-two (11%) of the participants had lacunes; 0 (0%) MA, 19 (15%) ischemic stroke (without migraine, *n* = 11; with migraine, *n* = 8), and 3 (8%) controls. Women with ischemic stroke had no increased risk of lacunes compared with controls, and there was no difference in the number of lacunes in stroke with and without migraine.

Three participants had focal cSS; all had ischemic stroke (one without migraine and two with migraine). More detailed information on microvascular damage is shown in Supplemental Table 3.

### Brain volume and cortical atrophy

**Figure 1.**
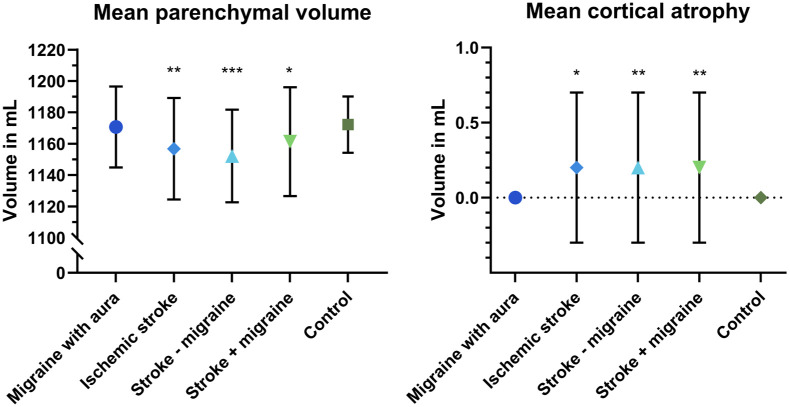
Mean parenchymal brain volume and mean cortical atrophy in mL. For all analyses, the control group was used as the reference group, and adjustment for age, hypertension, hypercholesterolemia, diabetes, and smoking was performed (*p < 0.05, **p < 0.01, ***p < 0.001).

Parenchymal brain volume and cortical atrophy were similar for MA compared with controls. Women with stroke had lower parenchymal brain volume compared with controls (overall: β = −19.6, 95% CI = −33.4 to −5.9; stroke with migraine: β = −14.2, 95% CI = −27.9 to −0.6; stroke without migraine: β = −25.2, 95% CI = −38.7 to −11.7, Table 3 and Figure 1). This difference was primarily found in stroke without migraine and was significantly different between stroke with and without migraine (*p* = 0.029, Supplemental Table 2). Women with stroke had increased cortical atrophy compared with controls (overall: β = 0.2, 95% CI = 0.0–0.4; stroke with migraine: β = 0.2, 95% CI = 0.0–0.4; stroke without migraine: β = 0.2, 95% CI = 0.1–0.4, Table 3 and Figure 1). There was no significant difference between stroke subgroups (*p* = 0.629, Supplemental Table 2).

**Table 3.** Linear regression of brain atrophy.

	β	95% CI	*p*
**Parenchymal volume**			
Migraine with aura	−0.9	−13.7 to 11.9	0.900
Ischemic stroke	−19.6	−33.4 to −5.9	**0.006**
*Stroke − migraine*	−*25.2*	−*38.7 to* −*11.7*	***0.000***
*Stroke* *+* *migraine*	−*14.2*	−*27.9 to* −*0.6*	***0.042***
**Cortical atrophy**			
Migraine with aura	0.0	−0.2 to 0.1	0.832
Ischemic stroke	0.2	0.0 to 0.4	**0.034**
*Stroke − migraine*	*0.2*	*0.1 to 0.4*	***0.001***
*Stroke* *+* *migraine*	*0.2*	*0.0 to 0.4*	***0.003***

For all analyses, the control group was used as the reference group. Adjusted for age, hypertension, hypercholesterolemia, diabetes, and smoking.

All significant *p*-values are depicted in bold.

*Italic:* the subgroups of women with stroke without migraine and women with stroke and migraine.

### Small vessel burden score

**Figure 2.**
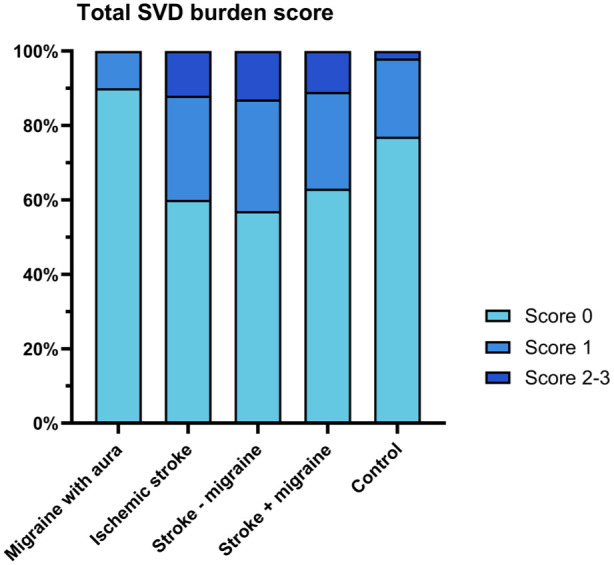
Total small vessel disease (SVD) burden scores across groups. The total SVD burden score was calculated by assigning one point for each of the following aspects: (1) perivascular WMH Fazekas score of 3 and/or deep WMH Fazekas score of 2–3, (2) presence of lacunes, (3) presence of microbleeds, and (4) presence of EPVS in basal ganglia with a score ⩾ 2. There was no difference in total SVD burden in MA or stroke compared with controls, and between stroke with and without migraine.

Across all groups, most participants had an SVD burden score of 0 (*n* = 142, 69%), 48 (23%) of 1, and 17 (8%) of 2 to 4 (Table 2 and Figure 2). Of the 48 participants who had score 1 for SVD burden, 15 (31%) had EPVS, 13 (27%) had cerebral microbleeds, 10 (21%) had WMH, and the remaining 10 (21%) had lacunes. There was no difference in total SVD burden in MA or stroke compared with controls, nor between stroke with and without migraine.

## Discussion

We found that markers of microvascular damage were infrequent in middle-aged women with MA and women without any neurological disease. In our exploratory study, women with a history of stroke had more cerebellar WMH, lower parenchymal volume, and more cortical atrophy compared with controls. We found no additive or supra-additive effect of migraine on microvascular damage in women with a history of stroke. These findings, however, need to be confirmed in larger cohorts.

A previous study showed that women with MA (mean age of 48 years) had an increased risk of deep WMH on 1.0–1.5T MRI.^3^ Although the clinical characteristics of MA patients from the CAMERA study are similar to those in our cohort, we could not confirm the increased risk for deep WMH in MA patients (without stroke) in our study. However, our sample size was smaller, and we used different measurements, MRI protocols, and scoring systems than in the CAMERA study, complicating direct comparison. Other more recent studies are in line with our current findings and did not show an increased risk for (deep) WMH in patients with MA.^25–27^

Although WMH are a frequent finding in patients with ischemic stroke,^28,29^ we did not find more WMH in women with ischemic stroke compared with controls. This could be due to our limited sample size, but this could also be due to the fact that our participants were younger than those in previous studies. However, we did found an increased presence of cerebellar WMH in women with stroke compared with controls. It remains unclear why WMH are increased in the cerebellum in these patients.

Furthermore, we found no differences in other markers of microvascular damage in women with MA compared with controls. Some older 0.5 to 1. 5T studies showed an association between EPVS and migraine (aura status unknown),^30,31^ whereas a recent 3T MRI study found an association with MO, but not with MA.^32^ In our 3T MRI study, we also found no association between EPVS and MA. Enlarged PVS are increased in lacunar stroke patients, but not other ischemic stroke subtypes.^11,33^ We indeed found that EPVS were not more frequent in women with ischemic stroke. We cannot exclude an association between EPVS and lacunar stroke because our study was not powered for such sub-analysis. We found cerebral microbleeds in women with ischemic stroke but rarely in women with MA or controls. cSS was only found in women after ischemic stroke and was probably due to hemorrhagic transformation of the infarcted area.

Previous studies found no association between MA (without genetic cause) and brain atrophy, which is in line with our findings.^34,35^ In patients with ischemic stroke, brain atrophy has been shown previously.^36,37^ These studies investigated total brain atrophy, including the stroke area. We now show that brain atrophy is present in middle-aged women with a history of ischemic stroke, even when only investigating the non-affected hemisphere.

Microvascular brain damage has multiple possible underlying causes, including blood–brain barrier damage, arterial stiffness, endothelial dysfunction, and inflammation.^8,38^ Furthermore, hypertension is often an exacerbating factor.^5^ Another important possible cause in patients with migraine, especially MA, is blood–brain barrier damage due to increased cortical spreading depolarization (CSD) susceptibility, which activates matrix-metalloproteinase-9.^39^ CSD is assumed to be the underlying cause of an aura and occurs especially in the occipital areas. Based on the frequency, location, or type of SVD markers in women with a history of MA or ischemic stroke, we found no clues that point toward a difference in underlying mechanisms of SVD between the two groups of women.

Our study has limitations. First, we included ischemic stroke patients with different underlying etiologies. The women with stroke without migraine more often had LAA, whereas the women with stroke with migraine more often had undetermined etiology. Next, not all stroke patients were analyzed for the presence of patent right-to-left shunting (patent foramen ovale (PFO)). Differences in group composition for stroke patients with and without migraine impair direct comparison. Second, the ischemic stroke group with migraine had either MO or MA, whereas the migraine group only had MA. Third, the MA and the control groups were relatively small; thus, we had limited power for analysis, which might explain why we did not find any differences between women with MA and controls. Another explanation might be a relatively high amount of microvascular damage in the control group and generally low frequencies of small vessel damage in the other groups. Fourth, selection bias might have occurred in the selection of MA patients, as these were retrieved from existing migraine cohorts from the LUMC hospital, which is a national tertiary headache center. These women are likely more conscious of their lifestyle and are under control of a medical professional, and might not be representative of the general migraine population.^40^ Last, due to the exploratory nature of the study and the small sample size, we were unable to adjust for more (rare) factors that could influence our results.

Our study also has strengths. We are the first to systematically assess a wide range of MRI markers for microvascular brain damage on high-quality 3T MRI scans in middle-aged women with or without stroke and migraine, while most previous studies focused on WMH only. Our study provides relevant new insights into the presence of the whole spectrum of microvascular damage in middle-aged women with MA, ischemic stroke, and a combination of both diseases. Our results are reassuring for women with MA and can be used to better inform middle-aged women in clinical practice about their risk of microvascular brain damage.

## Conclusion

Overall, markers of microvascular cerebral damage were uncommon in middle-aged women with MA and healthy controls, while a history of stroke might be associated with cerebellar WMH, lower parenchymal volume, and cortical atrophy. We found no (supra-)additive effect of migraine on microvascular brain damage in women with a history of stroke.

## Supplemental Material

sj-docx-1-wso-10.1177_17474930251389728 – Supplemental material for Microvascular brain damage in middle-aged women with a history of migraine with aura and/or ischemic stroke Supplemental material, sj-docx-1-wso-10.1177_17474930251389728 for Microvascular brain damage in middle-aged women with a history of migraine with aura and/or ischemic stroke by Nelleke van der Weerd, Annelise E Wilms, Hendrikus JA van Os, Ghislaine Holswilder, Katie M Linstra, Erik W van Zwet, Arn MJM van den Maagdenberg, Antoinette MaassenvandenBrink, Mark C Kruit, Gisela M Terwindt and Marieke JH Wermer in International Journal of Stroke

sj-docx-2-wso-10.1177_17474930251389728 – Supplemental material for Microvascular brain damage in middle-aged women with a history of migraine with aura and/or ischemic stroke Supplemental material, sj-docx-2-wso-10.1177_17474930251389728 for Microvascular brain damage in middle-aged women with a history of migraine with aura and/or ischemic stroke by Nelleke van der Weerd, Annelise E Wilms, Hendrikus JA van Os, Ghislaine Holswilder, Katie M Linstra, Erik W van Zwet, Arn MJM van den Maagdenberg, Antoinette MaassenvandenBrink, Mark C Kruit, Gisela M Terwindt and Marieke JH Wermer in International Journal of Stroke
