# Supplementary material for: Microvascular brain damage in middle-aged women with a history of migraine with aura and/or ischemic stroke
Source: Int J Stroke. 2025 Oct 15;21(5):699–707. doi: 10.1177/17474930251389728 (PMC13197609; doi:10.1177/17474930251389728)
Supplement: sj-docx-1-wso-10.1177_17474930251389728 – Supplemental material for Microvascular brain damage in middle-aged women with a history of migraine with aura and/or ischemic stroke [file sj-docx-1-wso-10.1177_17474930251389728.docx]

## Supplemental methods

## *MRI protocol*

MRI was performed with a Philips 3T MR scanner. The scanning protocol included 3D T1-weighted (repetition time (RT) = 8.2 ms, echo time (TE) = 4.5 ms, ACQ voxel MPS = 1.00x1.00x1.00), 2D T2-weighted (TR = 4783 ms, TE = 80 ms, ACQ voxel MPS = 0.43x0.50x3.00), 3D fluid-attenuated inversion recovery (FLAIR) (TR = 4800 ms, TE = 302 ms, inversion time = 1650 ms, ACQ voxel MPS = 1.12x1.12x1.12), and susceptibility weighted imaging (SWI) (TR = 45 ms, TE = 31 ms, ACQ voxel MPS = 0.78x0.79x1.60) scans.

***MRI markers of microvascular brain damage***

WMH were considered to be present if hyperintense on FLAIR, and divided in periventricular (PV-WMH), deep (D-WMH), and cerebellar. The Fazekas score was graded depending on the size and confluence.(1) The following scoring was used for PV-WMH: 0) absent, 1) caps or pencil-thin lining, 2) smooth halo, and 3) irregular periventricular signal extending into the deep white matter. The scoring for D-WMH was: 0) absent, 1) punctate foci, 2) beginning confluence, and 3) large confluent areas. Cerebellar WMH were only scored as present or absent.

Enlarges perivascular spaces (EPVS) were defined as fluid-filled spaces following vessels through grey or white matter. They were distinguished as being round or ovoid with a diameter <3 mm when imaged perpendicular to the vessel. EPVS were counted in the basal ganglia, centrum semiovale, and midbrain on T2-weighted scans following a qualitative rating scale.(2) Both hemispheres were counted, but only the hemisphere with the most EPVS was used. For midbrain, scoring was: 0) no EPVS visible, and 1) EPVS visible. For basal ganglia and centrum semiovale scoring was: 0) no EPVS, 1) 1-10 EPVS (mild), 2) 11-20 EPVS (moderate), 3) 21-40 EPVS (frequent), and 4) >40 EPVS (severe).

Cortical superficial siderosis (cSS) was scored on SWI images following two scoring systems: focal/disseminated or multifocality. cSS was rated as focal when it was restricted to 3 or fewer sulci and disseminated when it was affecting 4 or more sulci.(3) Multifocality scoring is done separately per hemisphere: 0) no cSS, 1) 1-3 sulci immediately adjacent with cSS, or 2) 2 or more non-adjacent sulci with cSS. The total score is of both hemispheres together (range 0-4): 0) no cSS, 1) mild and unifocal cSS, and 2-4) severe and multifocal cSS.(4) All areas of cSS were only scored if separated from hemorrhagic transformed ischemic stroke at least by 3 unaffected sulci.

Cerebral volume and cortical atrophy were determined using a fully automated tool cNeuro (version 1.11.0; Combinostics Ltd, Tampere, Finland). Cerebral volume was normalized for intra-cranial volume by (automatically) creating a loose brain mask to define the scaling factor. Volume was measured in mL, with 1 mL being 1000 mm^3^, and reflects the number of voxels multiplied by voxel size. Cortical atrophy was assessed according to the Pasquier visual rating scale (range 0-3),(5) and was computationally estimated based on the concentration of gray matter. Cortical atrophy ratings were classified as follows: 0) no atrophy, 1) mild atrophy with opening sulci, 2) moderate atrophy with volume loss of gyri, and 3) severe atrophy with knife blade atrophy. Both cerebral volume and cortical atrophy were determined in the unaffected hemisphere in the case of stroke patients. Cerebral volume was multiplied by two, to estimate total cerebral volume.

**References**

1. Fazekas F, Chawluk JB, Alavi A, Hurtig HI, Zimmerman RA. MR signal abnormalities at 1.5 T in Alzheimer's dementia and normal aging. AJR Am J Roentgenol. 1987;149(2):351-6.

2. Potter GM, Chappell FM, Morris Z, Wardlaw JM. Cerebral perivascular spaces visible on magnetic resonance imaging: development of a qualitative rating scale and its observer reliability. Cerebrovasc Dis. 2015;39(3-4):224-31.

3. Charidimou A, Linn J, Vernooij MW, Opherk C, Akoudad S, Baron JC, et al. Cortical superficial siderosis: detection and clinical significance in cerebral amyloid angiopathy and related conditions. Brain. 2015;138(Pt 8):2126-39.

4. Charidimou A, Boulouis G, Roongpiboonsopit D, Auriel E, Pasi M, Haley K, et al. Cortical superficial siderosis multifocality in cerebral amyloid angiopathy: A prospective study. Neurology. 2017;89(21):2128-35.

5. Pasquier F, Leys D, Weerts JG, Mounier-Vehier F, Barkhof F, Scheltens P. Inter- and intraobserver reproducibility of cerebral atrophy assessment on MRI scans with hemispheric infarcts. Eur Neurol. 1996;36(5):268-72.
