# Supplementary material for: Microvascular brain damage in middle-aged women with a history of migraine with aura and/or ischemic stroke
Source: Int J Stroke. 2025 Oct 15;21(5):699–707. doi: 10.1177/17474930251389728 (PMC13197609; doi:10.1177/17474930251389728)
Supplement: sj-docx-2-wso-10.1177_17474930251389728 – Supplemental material for Microvascular brain damage in middle-aged women with a history of migraine with aura and/or ischemic stroke [file sj-docx-2-wso-10.1177_17474930251389728.docx]

**Supplementary Table 1 Ordinal and logistic regression of microvascular damage markers (reference group: controls).**

|  | **OR** | **95%CI** | **p-value** |
| --- | --- | --- | --- |
| **WMH-PV** |  |  |  |
| Migraine with aura | 1.2 | 0.4–3.7 | 0.783 |
| Ischemic stroke | 1.1 | 0.4–3.5 | 0.839 |
| *Stroke – migraine* | *1.7* | *0.5–5.5* | *0.409* |
| *Stroke + migraine* | *1.1* | *0.3–3.7* | *0.785* |
| **WMH-D** |  |  |  |
| Migraine with aura | 1.2 | 0.4–3.3 | 0.792 |
| Ischemic stroke | 0.4 | 0.2–1.2 | 0.113 |
| *Stroke – migraine* | *0.6* | *0.2–1.8* | *0.350* |
| *Stroke + migraine* | *0.4* | *0.1–1.3* | *0.140* |
| **WMH cerebellar** |  |  |  |
| Ischemic stroke | 8.3 | 0.9–78.2 | 0.064 |
| *Stroke – migraine* | *11.7* | *1.2–111.9* | ***0.033*** |
| *Stroke + migraine* | *8.8* | *0.9–85.9* | *0.063* |
| **EPVS-BG** |  |  |  |
| Migraine with aura | 1.9 | 0.1–27.2 | 0.647 |
| Ischemic stroke | 3.1 | 0.3–34.0 | 0.350 |
| *Stroke – migraine* | *4.2* | *0.3–51.7* | *0.267* |
| *Stroke + migraine* | *5.5* | *0.5–65.8* | *0.174* |
| **EPVS-CS** |  |  |  |
| Migraine with aura | 0.6 | 0.2–1.5 | 0.281 |
| Ischemic stroke | 0.9 | 0.4–2.2 | 0.811 |
| *Stroke – migraine* | *0.9* | *0.3–2.3* | *0.825* |
| *Stroke + migraine* | *1.3* | *0.5–3.5* | *0.608* |
| **EPVS midbrain** |  |  |  |
| Migraine with aura | 2.3 | 0.8–6.9 | 0.145 |
| Ischemic stroke | 2.2 | 0.7–6.9 | 0.157 |
| *Stroke – migraine* | *2.6* | *0.8–8.2* | *0.113* |
| *Stroke + migraine* | *1.8* | *0.6–5.6* | *0.307* |
| **Cerebral microbleeds** |  |  |  |
| Ischemic stroke | 2.2 | 0.4–11.2 | 0.330 |
| *Stroke – migraine* | *2.0* | *0.4–10.8* | *0.434* |
| *Stroke + migraine* | *1.7* | *0.3–9.7* | *0.540* |
| **Lacunes** |  |  |  |
| Ischemic stroke | 2.2 | 0.4–13.7 | 0.343 |
| *Stroke – migraine* | *2.9* | *0.4–18.9* | *0.270* |
| *Stroke + migraine* | *1.7* | *0.3–11.6* | *0.570* |
| **Total SVD burden** |  |  |  |
| Migraine with aura | 0.5 | 0.1–1.8 | 0.269 |
| Ischemic stroke | 1.6 | 0.5–4.8 | 0.415 |
| *Stroke – migraine* | *2.1* | *0.7–6.5* | *0.211* |
| *Stroke + migraine* | *1.3* | *0.4–4.1* | *0.654* |

Adjusted for age, hypertension, hypercholesterolemia, diabetes, and smoking.

Controls were used as reference group.

Logistic regression for WMH cerebellar and ePVS midbrain.

Ordinal regression for SVD burden, WMH-PV, WMH-D, ePVS-BG, and ePVS-CS.

**Supplementary Table 2 Stroke subgroup analysis**

| **SVD marker** | **p-value** |
| --- | --- |
| WMH-PV | 0.374 |
| WMH-D | 0.456 |
| WMH cerebellar | 0.600 |
| ePVS-BG | 0.571 |
| ePVS-CS | 0.338 |
| ePVS midbrain | 0.421 |
| Microbleeds | 0.802 |
| Lacunes | 0.354 |
| Parenchymal volume | **0.029** |
| Cortical Atrophy | 0.629 |
| Total SVD burden | 0.239 |

Subgroup analysis where stroke with migraine and stroke without migraine were compared.

**Supplementary Table 3 Microvascular brain damage detailed score**

|  | **Migraine with aura**  **(n = 39)** | **Ischemic stroke**  **(n = 129)** | *Stroke – migraine*  *(n = 67)* | *Stroke + migraine*  *(n = 62)* | **Control**  **(n = 39)** |
| --- | --- | --- | --- | --- | --- |
| **WMH, ml ± sd** |  |  |  |  |  |
| WMH-PV* | 0.3 ± 0.3 | 0.9 ± 1.9 | *0.9 ± 2.1* | *0.9 ± 1.6* | 0.2 ± 0.2 |
| WMH-D* | 0.3 ± 0.6 | 0.3 ± 0.8 | *0.4 ± 1.0* | *0.2 ± 0.4* | 0.2 ± 0.6 |
| **EPVS^⸸^, n ± sd** |  |  |  |  |  |
| Basal ganglia | 6.3 ± 3.0 | 10.6 ± 5.4 | *11.7 ± 5.8* | *9.7 ± 4.9* | 6.7 ± 2.9 |
| Centrum semiovale | 13.7 ± 6.1 | 21.2 ± 9.6 | *23 ± 10.2* | *19.6 ± 8.8* | 15.8 ± 7.1 |
| Midbrain | 1.8 ± 1.7 | 2.2 ± 2.0 | *2.2 ± 2.1* | *2.1 ± 1.9* | 1.6 ± 1.5 |
| **Microbleeds**, n ± sd** | 0.0 ± 0.2 | 0.6 ± 2.7 | *0.4 ± 1.2* | *0.8 ± 3.7* | 0.2 ± 0.7 |
| **Lacunes*, n ± sd** | 0 ± 0 | 0.2 ± 0.7 | *0.3 ± 0.8* | *0.2 ± 0.6* | 0.1 ± 0.4 |

* Missing due to missing FLAIR MRI scan: WMH-PV (1), WMH-D (1), lacunes (1)

** Missing due to missing SWI MRI scan or moving artefacts: microbleeds (6)

**⸸** Missing due to missing T2 MRI scan or moving artefacts: BG (4), CS (10), midbrain (2)

**⸸⸸** Missing due to missing T1 MRI scan (2), atrophy data missing due to infarctions in both hemispheres (6)
